# A surgical instrument motion measurement system for skill evaluation in practical laparoscopic surgery training

**DOI:** 10.1371/journal.pone.0305693

**Published:** 2024-06-25

**Authors:** Koki Ebina, Takashige Abe, Lingbo Yan, Kiyohiko Hotta, Toshiaki Shichinohe, Madoka Higuchi, Naoya Iwahara, Yukino Hosaka, Shigeru Harada, Hiroshi Kikuchi, Haruka Miyata, Ryuji Matsumoto, Takahiro Osawa, Yo Kurashima, Masahiko Watanabe, Masafumi Kon, Sachiyo Murai, Shunsuke Komizunai, Teppei Tsujita, Kazuya Sase, Xiaoshuai Chen, Taku Senoo, Nobuo Shinohara, Atsushi Konno

**Affiliations:** 1 Graduate School of Information Science and Technology, Hokkaido University, Sapporo, Japan; 2 Department of Renal and Genitourinary Surgery, Faculty of Medicine, Hokkaido University, Sapporo, Japan; 3 Department of Gastroenterological Surgery II, Faculty of Medicine, Hokkaido University, Sapporo, Japan; 4 Clinical Simulation Center, Faculty of Medicine, Hokkaido University, Sapporo, Japan; 5 Department of Anatomy, Faculty of Medicine, Hokkaido University, Sapporo, Japan; 6 Faculty of Engineering and Design, Kagawa University, Takamatsu, Japan; 7 Department of Mechanical Engineering, National Defense Academy of Japan, Yokosuka, Japan; 8 Department of Mechanical Engineering and Intelligent Systems, Tohoku Gakuin University, Sendai, Japan; 9 Graduate School of Science and Technology, Hirosaki University, Hirosaki, Japan; Juntendo University School of Medicine Graduate School of Medicine: Juntendo Daigaku Igakubu Daigakuin Igaku Kenkyuka, JAPAN

## Abstract

This study developed and validated a surgical instrument motion measurement system for skill evaluation during practical laparoscopic surgery training. Owing to the various advantages of laparoscopic surgery including minimal invasiveness, this technique has been widely used. However, expert surgeons have insufficient time for providing training to beginners due to the shortage of surgeons and limited working hours. Skill transfer efficiency has to be improved for which there is an urgent need to develop objective surgical skill evaluation methods. Therefore, a simple motion capture–based surgical instrument motion measurement system that could be easily installed in an operating room for skill assessment during practical surgical training was developed. The tip positions and orientations of the instruments were calculated based on the marker positions attached to the root of the instrument. Because the patterns of these markers are individual, this system can track multiple instruments simultaneously and detect exchanges. However due to the many obstacles in the operating room, the measurement data included noise and outliers. In this study, the effect of this decrease in measurement accuracy on feature calculation was determined. Accuracy verification experiments were conducted during wet-lab training to demonstrate the capability of this system to measure the motion of surgical instruments with practical accuracy. A surgical training experiment on a cadaver was conducted, and the motions of six surgical instruments were measured in 36 cases of laparoscopic radical nephrectomy. Outlier removal and smoothing methods were also developed and applied to remove the noise and outliers in the obtained data. The questionnaire survey conducted during the experiment confirmed that the measurement system did not interfere with the surgical operation. Thus, the proposed system was capable of making reliable measurements with minimal impact on surgery. The system will facilitate surgical education by enabling the evaluation of skill transfer of surgical skills.

## 1 Introduction

In the field of abdominal or pelvic surgery, laparoscopic techniques are commonly used to avoid making large incisions on patients. In a laparoscopic operation, the surgeon inserts the endoscope and surgical instruments into the patient’s abdomen via a cannula and performs the procedure by referring to images from a camera. The smaller number of incisions made in laparoscopic surgery than in an open surgery results in lesser postoperative pain, smaller scars, and faster recovery. Additionally, the magnified field of view of the laparoscope enables precise surgical manipulation. These advantages have led to the wide use of laparoscopic surgery.

However, owing to the difficulties associated with laparoscopic surgery, surgeons must possess advanced skills. First, surgeons need to develop depth perception, which is the ability to transform a two-dimensional (2D) video image into a three-dimensional (3D) working area, because the operation is performed by referring to a 2D laparoscopic monitor. The narrower field of view also makes the operation more difficult compared with open surgery. Second, there are discrepancies in hand–eye coordination because the affected area cannot be visualized directly and needs to be referred to through a laparoscopic monitor. Third, surgeons must be accustomed to using specific surgical instruments that are located at a long distance from the handle to the tip. Because the surgical instrument moves around the cannula, which is the insertion point from the abdomen, the instrument’s operation is reversed between the handle and the tip owing to the fulcrum effect. Improper manipulation of the forceps can lead to complications such as organ damage and bleeding [1]. Hence, effective laparoscopic surgical training is required for beginners.

Several types of surgical training methods, such as dry labs, wet labs, and virtual reality (VR) surgical simulators, have been developed to impart and enhance surgical skills. Dry and wet labs provide surgical simulation training on human organ models made of plastic or rubber or cadaveric animal organs such as pigs. Although the operational feel is close to that of an actual operation in these training methods, the skills acquired are variable, and inappropriate habits may develop without proper guidance from a skilled surgeon [2]. VR surgical simulators have the advantage of providing training in a variety of situations, ranging from basic to practical. However, these surgical simulators are expensive and result in low-fidelity visual and haptic feedback [3]. Surgical training using live animals, human cadavers, and on-the-job training in clinical settings are also conducted as more practical surgery training. However, there are issues related to animal welfare, training opportunities, and medical safety. Because of the aforementioned technical proficiency challenges, the traditional apprenticeship model of “see one, do one, teach one” is inadequate for training surgeons in laparoscopic surgery. In addition, owing to the shortage of surgeons and limited working hours, expert surgeons have insufficient time for providing training and guidance to beginners. Therefore, there is an urgent need to develop an objective surgical skill evaluation method to improve the skill transfer efficiency.

To meet these demands, several studies on motion analysis and the assessment of surgeons’ psychomotor skills have been conducted in various training situations. Oropesa et al. [4] and Chmarra et al. [5] measured and analyzed the motion of surgical instruments for handling tasks such as pipe cleaners and peg transfer in a dry lab, respectively. Kowalewski et al. [6] and Franco-González et al. [7] performed motion analysis of needle drivers in the suturing/knotting task conducted in a dry lab. In a wet lab, Hofstad et al. [8] conducted cholecystectomy surgical measurements and an analysis of a porcine liver box model. In a cholecystectomy performed in a clinical setting, measurements of the surgeon’s hand motion using electromagnetic sensors were reported by Dosis et al. [9] and Rasmus et al. [10]. Hwang et al. [11] conducted forceps motion measurements using an optical MoCap system. A vision-based forceps motion measurement system was also developed and utilized to analyze the skills of surgeons [12, 13]. However, the surgical skill analysis in dry-lab training [4–7] focused only on fundamental laparoscopic procedures such as handling forceps and suturing/knotting. Although forceps motion measurements conducted during wet-lab training using a porcine cadaver organ [8] is more practical than that during dry-lab training, the training task did not involve forceps exchange, and the skills that could be measured were limited. The cholecystectomy surgical measurements in clinical settings reported by Dosis et al. [9] and Rasmus et al. [10] focused on the surgeon’s hand motions, and the movement of the surgical instruments was not measured. In the forceps motion measurements conducted by Hwang et al., the forceps’ tip position and operational force of grasping forceps were measured. However, this study measured only the grasping forceps, and the movements of other surgical instruments were not recorded. Furthermore, the motion of the forceps was restricted because they were connected to the measurement unit by cables; moreover, the measurement success rate and accuracy were not provided in this study. The cholecystectomy measurement conducted by Ganni et al. [12] measured only the 2D position of the instrument’s tip. Although a vision-based measurement system that can obtain 3D position of the instrument’s tip has been developed [4, 13] based on the principle of measurements, position measurements cannot be performed when the forceps are not visible in the operative field. In addition, because the positional data obtained using these systems are based on the local coordinate system of the laparoscope, the position and orientation of the scope must be recorded to transform the tip position into the world coordinate system. Accordingly, a surgical instrument motion measurement system has been developed by the authors in a previous study [14]. This system consists of six MoCap cameras that can track multiple surgical instruments and support instrument exchanges during surgery. Through the measurement experiment, 70 cases of lymphadenectomy and renal parenchyma suturing/knotting on a porcine cadaver organ were recorded, and the skill evaluation model was established [15–17]. However, the skill analysis during wet-lab training using pig cadavers was focused only on a simple procedure, which is insufficient for surgical education. Therefore, a simple MoCap-based measurement system that can be easily installed in an operating room was developed for skill assessment during practical surgical training [18]. Nevertheless, there are several obstacles in the operating room, and the measurement data include noise and outliers. Due to the simplification of the measurement system, the measurements were obtained only from the front of the surgeon; hence, the measurement accuracy was slightly inferior to that of the previous system. The effect of this decrease in measurement accuracy on feature calculation has to be determined.

Therefore, in this study, the post processing method for measurement data was newly developed, and the proposed system was further validated through the measurement experiment using animal organs and human cadavers. The results of the accuracy verification test and a comparison of the kinetic indices calculated from the measurement data of both our earlier systems are discussed. The development of outlier removal and smoothing methods are also reported. In addition, the measurement experiment results during cadaver surgical training and the questionnaire survey results regarding the operational feel while using the surgical instruments are presented. The validated system would help evaluate surgical skill and improve the skill transfer efficiency.

## 2 System configuration

### 2.1 Ethical statement

Approval was obtained from the Ethical Review Board for Life Science and Medical Research, Hokkaido University Hospital, for cadaveric anatomy and surgical training (CAST) for urologic surgery education and research (i16–043 issued on January 24, 2017), for skill evaluation in basic laparoscopic surgical procedures (ji018–0257 issued on March 12, 2019), and for observational study on measurement of forceps dynamics during laparoscopic surgery (i20–027) issued on December 16, 2020. Written informed consent was obtained from all participants for the use of data in the study. The cadaveric porcine tissues used in our measurement experiment were purchased from a commercial butcher. All procedures performed in studies involving human participants were in accordance with the ethical standards of the Institutional Research Committee and the Declaration of Helsinki of 1964 and its subsequent amendments or comparable ethical standards.

### 2.2 Measurement device

In this study, a trinocular MoCap camera unit (OptiTrack V120: Trio, NaturalPoint Inc.) was utilized as the measurement device [18]. The measurement system used in a previous study by the authors [14] consisted of six measurement cameras and required a large installation space, whereas the present system consists of only a trinocular camera unit and requires a smaller installation space. Because the three infrared (IR) cameras and IR light-emitting diodes (LEDs) were fixed in the unit, the positional relationship between the cameras does not change and no dynamic calibration is required. In this study, the measurement device was installed on top of the laparoscopic monitor using a dedicated installation bracket. Because the line of sight from the surgeon to the laparoscopic monitor is always guaranteed in laparoscopic surgery, stable measurement of surgical instruments is possible. These features make this system suitable for measurements in operating rooms with several obstacles.

### 2.3 Surgical instruments with markers

**Fig 1:**
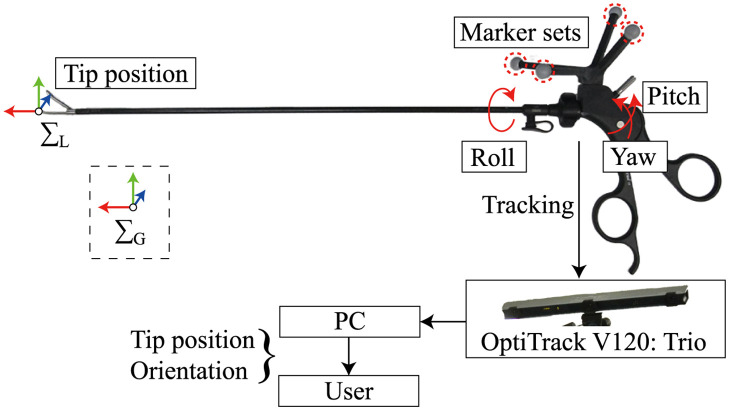
Overview of the grasping forceps with markers.

In laparoscopic surgeries, the tips of surgical instruments cannot be measured directly because they are inserted into the abdomen of the patient during the operation. Therefore, the proposed system calculates the tip position and orientation based on the positional relationship between the tip and the marker set attached to the root of the forceps. The marker set was attached to the surgical instrument using a dedicated jig made of ABS (acrylonitrile butadiene styrene)-like resin produced using a 3D printer. As each surgical instrument has a different marker pattern, the proposed system can recognize and track multiple surgical instruments and support intraoperative exchange. All surgical instruments used in the measurement experiments were equipped with a marker set. Fig 1 shows an overview of the grasping forceps with markers as representative examples.

### 2.4 Measurement data

**Fig 2:**
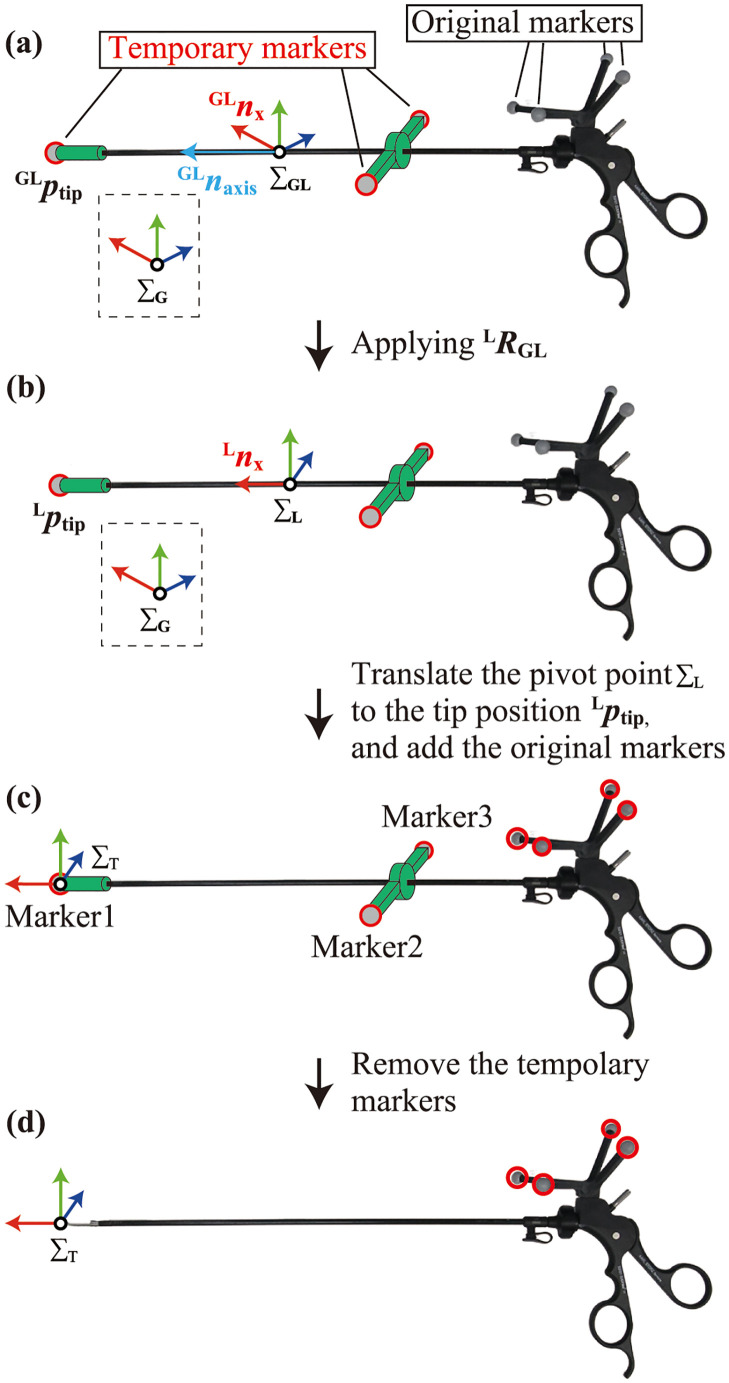
Registration process of the surgical instrument in the MoCap system.

The developed system measures the tip position and orientation of the surgical instruments. OptiTrack Motive 2.1.1 Final was used as control software for the MoCap system. By registering a marker set as a rigid body in the software, the arbitrary position and orientation of the rigid body can be obtained using its positional relationship with the barycenter of the marker set. Therefore, the tip position was defined by attaching a marker to the instrument tip; subsequently, the tip position can be calculated even if the tip marker is removed. The registration process of the tip position and orientation of the surgical instrument as follows:

(a) Temporary markers were attached to the sheath and tip of the instrument via dedicated marker jigs, and these markers were registered as a rigid body in MoCap software (see Fig 2a). Next, the centroid position of these markers was set as the pivot point of the rigid body, and the local coordinate system of the rigid body Σ_GL_ was defined with the same orientation as the global coordinate system. Notably, the marker set attached to the handle of the instrument was not registered. In the registration process, the surgical instruments were attached to the fixture such that the handles were vertical. This ensured that the y-axis of Σ_GL_ matched the orientation of the instrument’s handle.
(b) The sheath axis vector nGLaxis was obtained from the tip marker position pGLtip in the local coordinate system of the rigid body. As the structure of the temporary jig in which the two beams had the same length, the centroid position of the rigid body was located on the sheath axis; therefore, the following equality holds:
  nGLaxis=pGLtip||pGLtip||. (1)
(c) The rotation matrix RLGL was calculated such that the sheath axis nGLaxis and the x-axis of the rigid body nGLx coincided based on Rodrigues’ rotation formula as follows:
  RLGL=cosθI+(1-cosθ)uuT+sinθ[u]×, (2)
  where ***I*** is the identity matrix; ***u*** is the axis of rotation, *θ* is the rotation angle; and [***u***]_×_ is the skew-symmetric cross-product matrix of ***u***. These values are calculated as follows:
  u=nGLx×nGLaxis||nGLx×nGLaxis||, (3)
  θ=cos-1nGLx·nGLaxis||nGLx||||nGLaxis||, (4)
  $$\begin{array}{c} {\left[u\right]}_{\times }=[\begin{array}{ccc} 0 & -u_{z} & u_{y}\\ u_{z} & 0 & -u_{x}\\ -u_{y} & u_{x} & 0 \end{array}], \end{array}$$ (5)
  where nGLx=(1,0,0), ***u*** = (*u*_*x*_, *u*_*y*_, *u*_*z*_). After the calculation, RLGL was transformed to Z-Y-X Euler angles and inputted to the MoCap software. Once the rotation angles were inputted, these angles were recorded and the MoCap software automatically applied a rotation such that the sheath axis and x-axis of the rigid body were coincident (Fig 2b).
(d) The pivot point of the rigid body Σ_L_ was translated to the tip marker position pLtip in the MoCap software, and the original markers were added to the rigid body (Fig 2c).
(e) The temporary markers were removed (Fig 2d). Subsequently, the tip position and orientation of the instrument were calculated only with the handle markers.

In this system, the tip position was obtained from the global coordinate system Σ_G_ (see Fig 1), and the orientation was obtained in a quaternion form.

## 3 Accuracy verification test

As the developed system calculates the tip position of the instrument by measuring the markers attached to the root, the positional error is amplified by the leverage effect. Therefore, an accuracy verification test was conducted in the previous study [18] by comparing the measurement data obtained from this system with those obtained using the former system [14]. The experiment was conducted during wet-lab training using a cadaveric porcine organ. The participants performed a dissection and suturing/knotting task.

### 3.1 Participants and tasks

**Fig 3:**
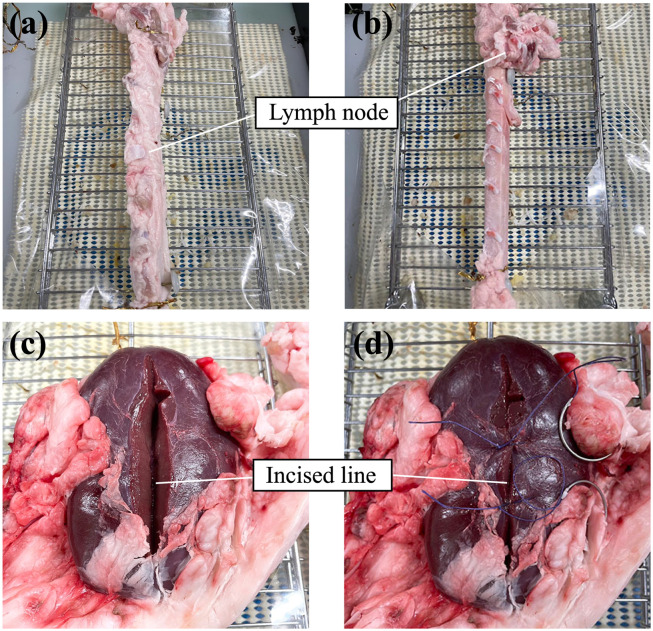
Overview of the porcine cadaver organ. (a),(b) An aorta of porcine cadaver before (a) and after (b) the dissection task (c),(d) Incised kidney of porcine cadaver before (c) and after (d) the suturing/knotting task.

The experimental task involved the dissection of lymph nodes and suturing/knotting of the renal parenchyma in a cadaveric porcine organ. In the dissection task, the participants were asked to detach the lymph node from the aorta using three surgical instruments: grasping forceps, scissor forceps, and a clip applier (Weck Hem-o-lok Polymer Locking Ligation System Applier; size L). In the suturing/knotting task, the participants were asked to suture the incised kidney at two sites using two needle holders. Both tasks have been reported to have high construct validity [19] and have been performed in previous studies [15–17]. An overview of the cadaveric porcine organs used in each task is presented in Fig 3. Fifteen surgeons (13 urologists and 2 junior residents) participated in the experiment. As one urologist participated twice, 16 measurement data were obtained. The background of the participant’s is presented in S1 Table. As presented in the table, a left-handed participant was included in the experiment. However, the data of this participant were not excluded from the analysis in our previous studies [18] because the participant performed the surgery right-handedly. The study was approved on March 12, 2019 (ji018–0257), and the experiments were conducted from August 7 to August 18, 2020.

### 3.2 Experimental environment

**Fig 4:**
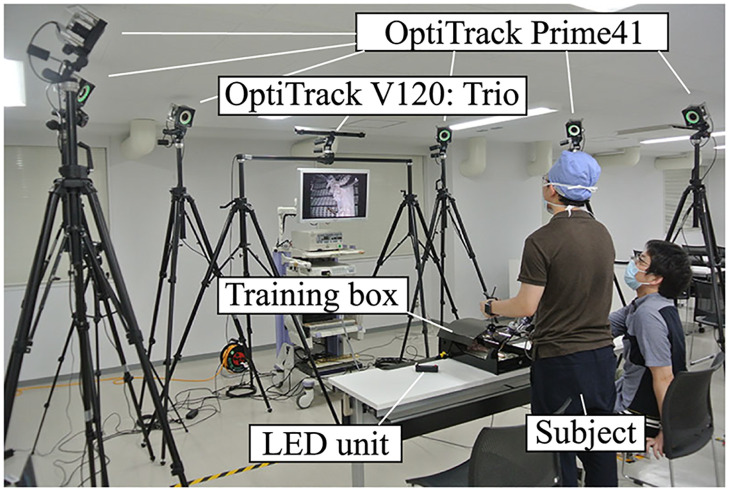
Experimental environment.

**Fig 5:**
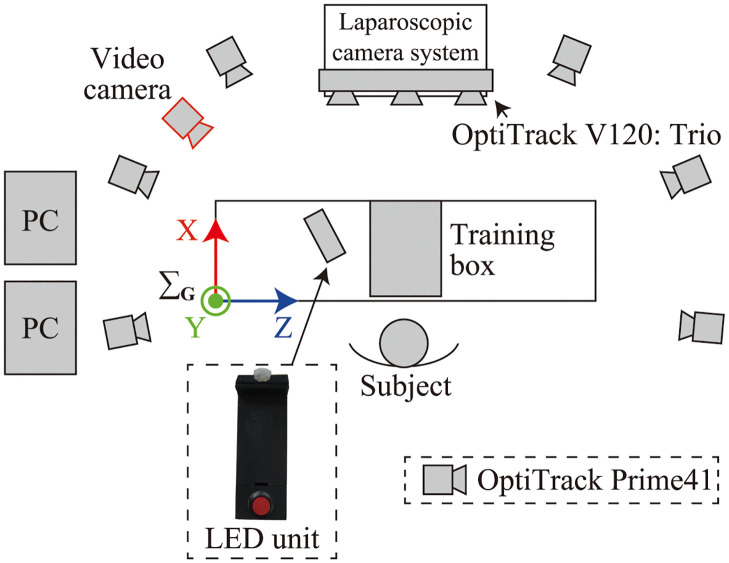
Camera configuration.

To verify the accuracy of the measurement system, the optical MoCap system OptiTrack Prime41 was used. In the experiment, six OptiTrack Prime41 cameras were set to surround the participant, and the OptiTrack V120: Trio was placed above the laparoscopic monitor. Fig 4 shows the experimental environment, and Fig 5 illustrates the camera configuration. Each system independently measured the movement of the surgical instruments. The measurement success rate and accuracy of the measurement data obtained using OptiTrack V120: Trio were validated by comparing them with those obtained using OptiTrack Prime41 as true values. The measurement frequency was 120 Hz for both systems and the measured data were smoothed using a Savitzky–Golay filter (window size:121, polynomial order:3) [20]. Missing position data owing to occlusion and marker interference were interpolated by linear interpolation, and the orientation data were interpolated in a quaternion form by spherical linear interpolation (Slerp).

### 3.3 Synchronization

An IR LED signal unit (Fig 4) was used to synchronize the two measurement systems; synchronization was performed manually after the experiment based on the timing of the detection of the LED signal. In some cases, LED signals could not be detected; thus, manual synchronization was performed based on the instrument’s movement measurement data. The global coordinate systems of the two measurement systems were matched using the same calibration square when the origin of the coordinate system was set.

### 3.4 Results

Table 1 presents the experimental results for each surgical instrument. *A*_prime41_ and *A*_trio_ are the measurement success rates of OptiTrack Prime41 and OptiTrack V120: Trio, respectively. $\bar{\Delta p}$ and $\bar{\Delta \theta }$ are the positional and attitude angle errors of the developed system. In the dissection task, the measurement data of the scissor forceps were excluded from verification because of a defect in the marker jig of the forceps. In the validation, the measurement data were analyzed while the tip of the instrument was moving inside the training box; the data when the tip was moving outside the box were excluded. The measurement success rate *A* was calculated as follows:

$$\begin{array}{c} A=100\frac{N_{\text{captured}}}{N}\;\;\left(\%\right), \end{array}$$ (6)

where *N*_captured_ is the number of frames successfully captured by OptiTrack Prime41 or V120: Trio, and *N* is the total number of frames in the task. As noted above, the frames in which surgical instruments were not used were excluded. The Wilcoxon signed rank sum test, a nonparametric paired statistical test, was also used to evaluate the difference in the measurement success rates between the two systems. The test result is presented in Table 1, and the box plot of the success rate of the measurement is shown in S1 Fig. The mean position error $\bar{\Delta p}$ of OptiTrack V120: Trio was calculated as follows:

$$\begin{array}{c} \bar{\Delta p}=\frac{1}{N_{\text{captured}}}\sum_{i=1}^{N_{\text{captured}}}\left|\right|p_{\text{ref}}-p_{\text{observed}}\left|\right|\;\;\left(\text{mm}\right), \end{array}$$ (7)

where ***p***_**ref**_(*i*) and ***p***_**observed**_(***i***) are the instrument tip positions in frame *i* measured using OptiTrack Prime41 and OptiTrack V120: Trio, respectively. In this study, the mean attitude angle error was calculated using the rotation angle of the differential quaternion. The mean attitude angle error $\bar{\Delta \theta }$ was calculated as follows:

$$\begin{array}{c} \bar{\Delta \theta }=\frac{360}{2\pi }\frac{1}{N_{\text{captured}}}\sum_{i=1}^{N_{\text{captured}}}2\;{\text{cos}}^{-1}\;q_{w}\left(i\right)\;\;\left({}^{\circ}\right), \end{array}$$ (8)

where *q*_*w*_ denotes the element of the difference quaternion Δ***Q*** = (*q*_*w*_, *q*_*x*_, *q*_*y*_, *q*_*z*_). The difference quaternion Δ***Q*** is expressed as follows:

$$\begin{array}{c} \Delta Q=Q_{\text{observed}}^{-1}Q_{\text{ref}}, \end{array}$$ (9)

where *Q*_observed_ and *Q*_ref_ are the quaternions measured by OptiTrack V120: Trio and OptiTrack Prime41, respectively.

**Table 1:** Measurement success rate and average error of each surgical instrument.

	*A*_prime41_ (%)	*A*_trio_ (%)	*p*-value	$\bar{\Delta p}$ (mm)	$\bar{\Delta \theta }$ (°)
Grasping forceps	99.99 (99.96–100.00)	98.83 (98.01–99.38)	< 0.001	3.28 (1.95–4.22)	0.86 (0.53–0.92)
Clip applier	100.00 (99.99–100.00)	99.38 (98.39–99.83)	< 0.001	4.42 (2.71–9.37)	1.43 (0.79–2.40)
Needle holder (R)	100.00 (99.99–100.00)	86.73 (81.34–91.70)	< 0.001	2.76 (2.58–2.95)	1.15 (1.02–1.41)
Needle holder (L)	99.99 (99.76–100.00)	94.48 (86.21–97.27)	< 0.001	2.56 (2.27–3.21)	1.01 (0.83–1.72)

### 3.5 Comparison of the kinematic indices between the systems

In this study, as the developed system is intended for evaluating the skill of the surgeon, the kinematic indices calculated from the measurement data of the developed system were also verified. The summary of these indices as follows:

(a) Path length: PL (m)
(b) Depth path length: DPL (m)
(c) Depth velocity: DV (cm/s)
(d) Percentage of PL in close zone (0 ≤ to < 2.0 (cm) from target object): Close (%)
(e) Average velocity: $\bar{v}\left(\text{cm}/\text{s}\right)$
(f) Average acceleration: $\bar{a}\left(\text{cm}/{\text{s}}^{2}\right)$
(g) Average jerk: $\bar{j}\left(\text{cm}/{\text{s}}^{3}\right)$
(h) Time percentage of forceps moving at certain velocity range
  (i) Idle (%) [0 ≤ to < 0.5 (cm/s)]
  (ii) Low (%) [0.5 ≤ to < 2.0 (cm/s)]
  (iii) Middle (%) [2.0 ≤ to < 5.0 (cm/s)]
  (iv) High (%) [5.0 ≤ to < 12.0 (cm/s)]
(i) Angular length along roll: AL-Roll (°)
(j) Angular length along pitch and yaw: AL-PitchYaw (°)

The calculated indices were defined and utilized for skill evaluation in the previous studies by the authors [15–17]. These indices were calculated from the measured data of both systems, and Spearman’s rank correlation coefficients were calculated to compare the values. Table 2 lists the results of the verification of kinematic indices. The indices not used in skill evaluation in the previous studies were excluded from the calculation.

**Table 2:** Spearman’s rank correlation coefficients of kinematic indices between both systems.

	PL	DPL	DV	Near	v	a	j	Idle	Low	Middle	High	AL-Roll	AL-PitchYaw
Grasping forceps	0.982	0.843	0.943	0.707	0.968	0.961	0.946	0.968	0.971	0.989	0.968	0.968	0.989
Clip applier	0.929	0.921	0.936	0.750	0.946	0.771	0.725	0.911	0.854	0.886	0.875	0.701	0.854
Needle holder (R)	0.957	0.968	0.961	0.786	0.989	0.986	0.986	0.961	0.957	0.961	0.975	0.989	0.982
Needle holder (L)	0.986	0.989	0.996	0.836	0.971	0.968	0.950	0.936	0.975	0.918	0.996	0.979	0.971

### 3.6 Questionnaires regarding the operational feel of the instruments

After conducting the experiment, a questionnaire survey regarding the operational feel of the surgical instruments was conducted to investigate whether the markers attached to the forceps interfered with the surgical procedure. The participants were asked to respond regarding the operating feel of the five surgical instruments used in the training task on a 5-point scale (1: I do not think that it was disturbing at all; 2: I do not think that it was disturbing; 3: Neutral; 4: I agree that it was disturbing; and 5: I strongly agree that it was disturbing). The questions were as follows: Did the markers attached to the instruments interfere with the operation?

(a) Grasping forceps
(b) Scissor forceps
(c) Clip applier
(d) Needle holder (R)
(e) Needle holder (L)

Responses were obtained from 15 subjects. For the subjects who participated twice, only the first response was used in the analysis. A summary of the questionnaire is presented in Table 3.

**Table 3:** Averages of user evaluation of surgical instruments with markers.

	Evaluation					Average scores
	1	2	3	4	5	
Grasping forceps	7	7	1	0	0	1.60
Scissor forceps	7	7	1	0	0	1.60
Clip applier	12	3	0	0	0	1.20
Needle holder (R)	11	4	0	0	0	1.27
Needle holder (L)	11	3	0	1	0	1.40

## 4 Measurement experiments during surgical training on cadavers

Measurement experiments were then conducted during surgical training on cadavers to investigate the measurement stability and establish a measurement system in the operation room of training. A donated human cadaver is generally used for surgical training, by which surgeons can improve their skills on actual anatomical structures. This form of surgical training is regularly conducted twice a year by the Department of Renal and Genitourinary Surgery, Graduate School of Medicine, Hokkaido University, to improve the practical surgical skills of surgeons. For this training, the donated cadaver body is fixed using the Thiel method [21] such that the properties of the cadaver body, such as the color and soft tissues, are almost identical to those of a living body. Therefore, a high educational level can be expected because the surgery can generally be performed as in a real case, except for some factors such as the absence of bleeding. In these experiments, 31 urologists performed laparoscopic radical nephrectomy and the movement of the surgical instrument was recorded using the proposed system.

### 4.1 Participants and task

The CAST was approved on January 24, 2017 (i16–043), and the study was approved on December 16, 2020 (i20–027). A total of 31 urologists were included in the study. Because five urologists participated in the experiment twice, 36 cases of measurement data were obtained. The background of the participants is presented in S2 Table. Although a left-handed participant was enrolled in the experiment, the corresponding data were not excluded from the analysis because the subject performed the actual surgery right handedly. The experiments were conducted in five terms from January 11, 2021 to January 10, 2023 (1st: January 11 to January 22, 2021, 2nd: July 27 to August 19, 2021, 3rd: November 4, 2021 to January 8, 2022, 4th: September 6 to September 16, 2022, 5th: January 5 to January 10, 2023). The training task in this experiment was laparoscopic radical left/right nephrectomy via a transperitoneal approach. During the training, the surgeon inserted the surgical instruments through three to five locations in the abdomen using cannulas. The surgeon performed only one left or right nephrectomy in each experiment. The cadaver was placed in the left or right lateral position, depending on the kidney to be removed. After inserting the cannula to be used as the laparoscopic camera port, carbon dioxide insufflation was performed. The task began with the insertion of two cannulas into the abdomen and was completed when the kidneys were dissected from the surrounding tissue, except for the ureters. In addition to the grasping forceps, scissors forceps, and clip appliers, Ligasure (Covidien LigaSure Maryland37NC), right-angle dissecting forceps (manufactured by OLYMPUS), and suction instruments (Covidien SurgiwandII) were used in the task.

### 4.2 Experimental setup

**Fig 6:**
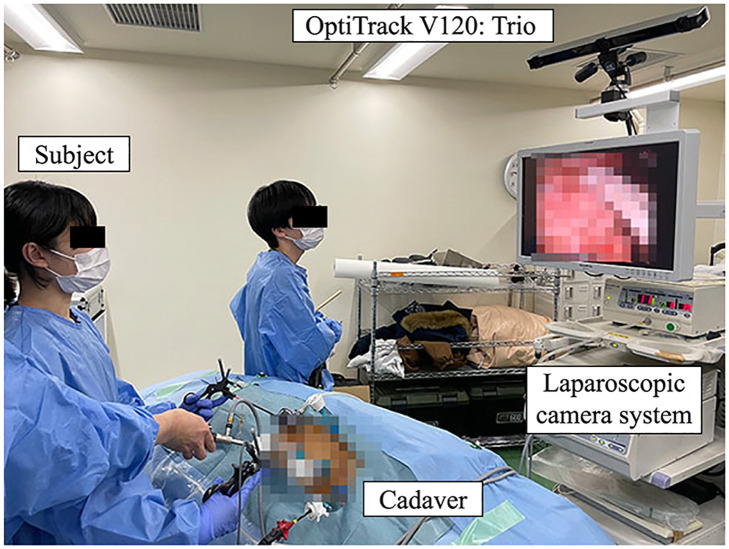
Experiment environment.

**Fig 7:**
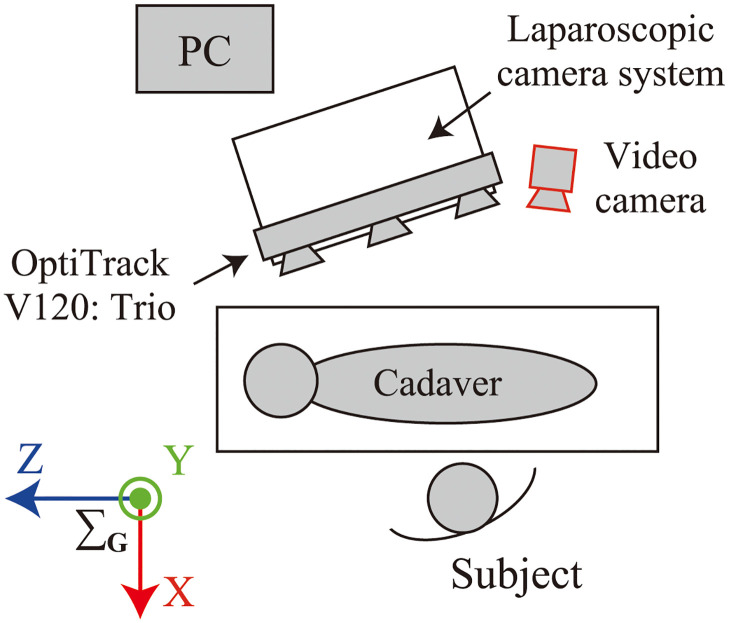
Experimental device set up.

**Fig 8:**
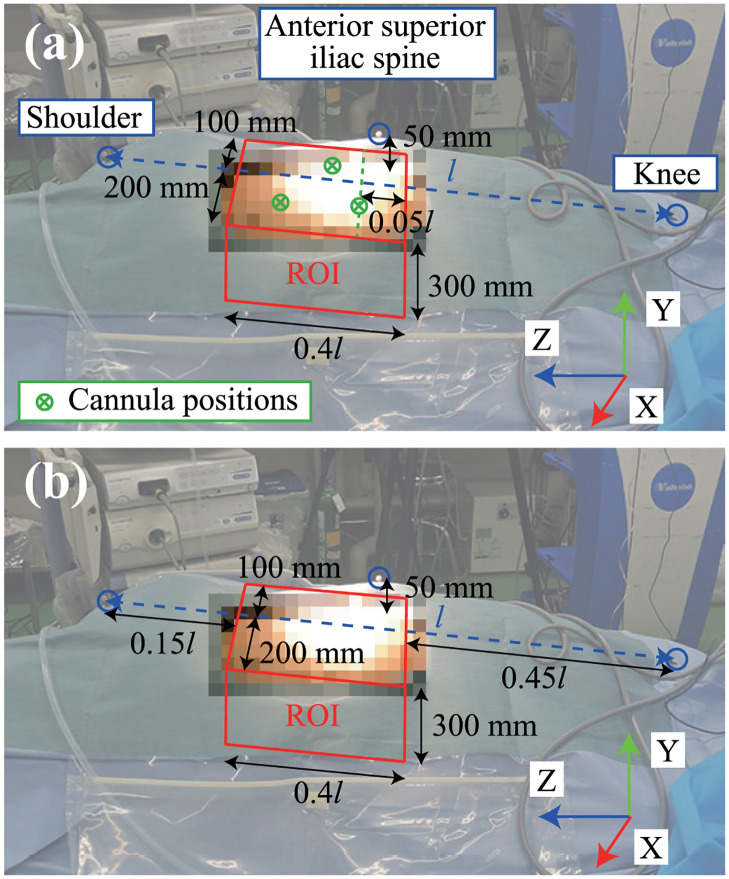
Definition of ROI. (a) ROI based on cannula position. (b) ROI based on anatomical marker position.

The experimental environment and experimental device setup are shown in Figs 6 and 7, respectively. The entire training period was recorded using a video camera, as shown in Fig 7, as well as using a laparoscopic camera. The measurement settings and parameters were the same as those used in the accuracy verification tests. Because the measurement data contained considerable noise and outliers due to the various obstacles in the room, these were removed by performing data processing described in the next section. After insufflation was completed and all cannulas were inserted, the vinyl portion of the surgical drape over the cadaver was closed because the measurements were strongly affected by reflective objects. IR markers were attached to three anatomical features, namely, the shoulder, anterior superior iliac spine, and knee, to determine the relationship between the surgical instruments and the cadaver body (see Fig 8). The port positions at which the cannula was inserted into the abdomen were recorded using IR markers after the experiment. In this study, a region of interest (ROI) was defined based on the cannula positions, and the frames during the tip of the instrument not in the ROI on the abdomen were excluded from the analysis. Because the cannula positions were not recorded in the five experiments, the ROI based on the anatomical marker positions shown in Fig 8b was used in the experiments. Both ROIs were of the same size and their validity was visually confirmed from the plots of the surgical instrument tip positions for all data.

### 4.3 Results

Among the 36 cases in the experiment, 9 failed owing to malfunctions of the hardware of the measurement equipment, and 2 were excluded from the analysis owing to inadequate cadaver body conditions. Therefore, measurement data from 25 cases were obtained for skill analysis. The malfunction of the measurement system was resolved by replacing the PC. Therefore, this was not considered a major challenge in the system. Table 4 and S2 Fig lists the measurement success rates for each surgical instrument used in the experiment. Note that the measurement success rate was calculated using Eq 6.

**Table 4:** Summary of the measurement success rate (%) in cadaver surgical training.

	*A*_trio_
Grasping forceps	92.74 (89.38–96.26)
Scissors forceps	92.27 (87.68–95.75)
Clip applier	99.39 (94.08–99.95)
Suction instrument	97.63 (94.62–98.95)
Ligasure	93.85 (87.59–97.67)
Right angle forceps	98.45 (94.58–99.92)

Median (1st quartile–3rd quartile)

### 4.4 Questionnaires regarding the operational feel of the instruments

The influence of the marker set attached to the surgical instruments was investigated using a questionnaire on the feel of the operation. The participants were asked to respond on the operational feel of the six surgical instruments used in the training task on a 5-point scale (1: I do not think that it was disturbing at all; 2: I do not think that it was disturbing; 3: Average; 4: I agree that it was disturbing; and 5: I strongly agree that it was disturbing). The questions were as follows: Whether the markers attached to the instruments interfered with the operation.

(a) Grasping forceps
(b) Scissor forceps
(c) Clip applier
(d) Suction instrument
(e) Ligasure
(f) Right angle forceps

Responses were obtained from 31 subjects. Five subjects participated twice, but only the first response was used in the analysis. A summary of the results of this survey is shown in Table 5. In addition to the 25 successfully conducted experiments, questionnaires were collected from 11 experiments that failed or were excluded from the analysis because of the poor conditions of the cadaver body. Responses were collected regarding the surgical instruments used in the task, and unused surgical instruments were excluded.

**Table 5:** Averages of user evaluation of surgical instruments with markers in cadaver surgical training.

	Evaluation					Average scores
	1	2	3	4	5	
Grasping forceps (*n* = 31)	19	5	3	4	0	1.74
Scissor forceps (*n* = 31)	16	5	2	8	0	2.06
Clip applier (*n* = 30)	21	6	1	2	0	1.47
Suction instrument (*n* = 17)	13	3	1	0	0	1.29
Ligasure (*n* = 30)	18	8	2	2	0	1.60
Right angle forceps (*n* = 27)	20	6	1	0	0	1.30

## 5 Data correction process for noise and outliers

As described in the previous section, a high success rate was achieved in the measurement experiment. However, because this system only measures instrument motion from one direction, the measurement data often include noise and outliers owing to misrecognition by the MoCap system. In this study, the data processing method for removing noise and outliers was evaluated. The smoothing and interpolation processes for the missing values are described in this section.

### 5.1 Outlier removal method

#### 5.1.1 Outlier removal based on the partial measurement success rate

Measurement data often include burst errors or missing data of several seconds owing to marker occlusion or overlapping of the markers. The measurement data obtained under such unstable conditions contain several outliers and should be excluded for a reliable skill analysis. Therefore, a threshold-based outlier removal method for measuring the success rate was implemented. In this method, the partial measurement success rate (PMR) was calculated using a moving window and data were removed when the PMR fell below a threshold value. The moving window size was set to 600 frames (5 s), the reference point of the window was set at the center, and the threshold of PMR was set to 25%. Therefore, in this study, the PMR was calculated based on the frames 5 s before and after the frame to be evaluated; if the rate was less than 25%, the frame was eliminated.

#### 5.1.2 Outlier removal based on the ROI

When a misrecognition occurs in the MoCap system, the tip position of the surgical instrument traverses the ROI defined in Fig 8 for a short period. Hence, the data removal process for the data for which measurement was not conducted for a certain period after moving from the inside to the outside of the ROI was performed. The threshold for the continuous measurement was set to 5s. The missing values with a duration of less than 0.1s were ignored in this process for noise reduction. By applying this process, outliers that were not outside the ROI owing to misrecognition by the MoCap system could be excluded.

#### 5.1.3 Outlier removal based on the estimated values of the Kalman filter

In this study, the outlier removal method based on the estimated value of the Kalman filter was implemented. The Kalman filter is a type of sequential Bayesian filter that models the target system and estimates its state from measurement data. In this process, the measured data are compared with the estimated value of the Kalman filter, and, if the discrepancy between the two values exceeds the threshold, the measured data is considered an outlier and removed. The second-order Kalman filter was generated for the tip position of the surgical instrument by setting the linear discrete-time state equation and the observation equation. Let $x\left(k\right)={\left[\begin{array}{ccc} p(k) & \dot{p}\left(k\right) & \ddot{p}\left(k\right) \end{array}\right]}^{T}$ for the state variables and $z\left(k\right)={\left[\begin{array}{c} \bar{p}\left(k\right) \end{array}\right]}^{T}$ for the observed variables. The state and observation equations are defined as follows:

x(k)=Fx(k-1)+Γv(k) (10)

z(k)=Hx(k)+w(k), (11)

***F*** in Eq 10 is a state transition matrix of size 9×9 and **Γ** is the noise gain of the system. The matrices are defined as follows:

$$\begin{array}{c} F=[\begin{array}{ccc} I & \Delta tI & \frac{1}{2}{\Delta t}^{2}I\\ O & I & \Delta tI\\ O & O & I \end{array}], \end{array}$$ (12)

$$\begin{array}{c} \Gamma ={\left[\begin{array}{ccccccccc} \frac{1}{2}\Delta t^{2} & \frac{1}{2}\Delta t^{2} & \frac{1}{2}\Delta t^{2} & \Delta t & \Delta t & \Delta t & 1 & 1 & 1 \end{array}\right]}^{T}. \end{array}$$ (13)

where Δ*t* is the sampling period of the MoCap system (Δ*t* = 1/120(*s*)). ***H*** in Eq 11 is the observation matrix, defined as follows:

$$\begin{array}{c} H=[\begin{array}{cc} I & O_{3\times 6} \end{array}] \end{array}$$ (14)

***v***(*k*) and ***w***(*k*) in Eqs 10 and 11 are normal white noise with a mean of 0 and variances of $\sigma_{v}^{2}$ and $\sigma_{w}^{2}$, which are called system noise and observation noise, respectively. Notably, ***v***(*k*) and ***w***(*k*) are independent of each other. In this study, $\sigma_{v}^{2}$ and $\sigma_{w}^{2}$ were set to $\sigma_{v}^{2}=0.01\left({\text{m}}^{2}\right)$ and $\sigma_{v}^{2}=0.0001\left({\text{m}}^{2}\right)$, respectively. Based on these parameters, the state estimation was conducted as follows:

**Estimation step**
$${\hat{x}}^{-}\left(k\right)=A\hat{x}\left(k-1\right),$$ (15)
$$P^{-}\left(k\right)=AP\left(k-1\right)A^{T}+\sigma_{v}^{2}\Gamma \Gamma^{T},$$ (16)
  where ${\hat{x}}^{-}\left(k\right)$ is the prior state estimate and ***P***^−^(*k*) is the prior error covariance matrix. The state estimate $A\hat{x}\left(k-1\right)$ and the posterior error covariance matrix ***P***(*k* − 1) at frame *k* − 1 are calculated using the following update step.
**Update step**
$$G(k)=\frac{P^{-}\left(k\right)H^{T}}{{HP}^{-}\left(k\right)H^{T}+\sigma_{w}^{2}},$$ (17)
$$\hat{x}\left(k\right)={\hat{x}}^{-}\left(k\right)+G\left(k\right)\left\{z\left(k\right)-H{\hat{x}}^{-}\left(k\right)\right\},$$ (18)
$$P(k)=S\left(k\right)P^{-}\left(k\right)S{\left(k\right)}^{T}+\sigma_{w}^{2}G\left(k\right)G{\left(k\right)}^{T},$$ (19)
  where ***S***(*k*) = ***I*** − ***G***(*k*)***H***. ***G***(*k*) is the Kalman gain, $\hat{x}\left(k\right)$ is the state estimate, and ***P***(*k*) is the posterior error covariance matrix. The posterior error covariance matrix ***P***(*k*) is generally obtained by ***P***(*k*) = ***I*** − ***G***(*k*)***HP***^−^(*k*); however, rounding errors in numerical calculations on a computer can cause asymmetry, which may lead to problems such as divergence of the Kalman filter. Therefore, Joseph’s equation [22] expressed in Eq 19 was used in this study.

In this study, the outliers were detected by comparing the prior state estimates ${\hat{x}}^{-}\left(k\right)$ obtained using Eq 15 with the measured value ***z***(*k*) in the prediction step. First, ***a***(*k*) is obtained from the prior state estimate ${\hat{x}}^{-}\left(k\right)$ as the estimated value of the tip position of the surgical instrument using the Kalman filter at frame *k* as follows:

$$\begin{array}{c} a\left(k\right)={\left[\begin{array}{ccc} {\hat{x}}_{1}^{-}\left(k\right) & {\hat{x}}_{2}^{-}\left(k\right) & {\hat{x}}_{3}^{-}\left(k\right) \end{array}\right]}^{T}, \end{array}$$ (20)

where ${\hat{x}}_{i}\left(k\right)$ is the *i*th element of $\hat{x}\left(k\right)$. From the prior error covariance matrix ***P***^−^(*k*) in Eq 16, let the *i* and *j* elements of ***P***^−^(*k*) be denoted as $p_{ij}^{-}\left(k\right)$, and the variances of the state variables $\sigma_{x}^{2},\sigma_{y}^{2}$, and $\sigma_{z}^{2}$ can be obtained by $p_{11}^{-}\left(k\right),p_{22}^{-}\left(k\right)$, and $p_{33}^{-}\left(k\right)$,respectively. Thus, the standard deviations σ(k) corresponding to the state variables *σ*_*x*_, *σ*_*y*_, and *σ*_*z*_ are expressed as follows:

$$\begin{array}{c} \sigma \left(k\right)={\left[\begin{array}{ccc} \sqrt{p_{11}^{-}\left(k\right)} & \sqrt{p_{22}^{-}\left(k\right)} & \sqrt{p_{33}^{-}\left(k\right)} \end{array}\right]}^{T}. \end{array}$$ (21)

Therefore, when ***a***(*k*) satisfies the following equation, the observed value ***z***(*k*) is handled as an outlier and the update step is not performed:

$$\begin{array}{c} ||a\left(k\right)-z\left(k\right)||>r_{\text{th}}||\sigma \left(k\right)||, \end{array}$$ (22)

where *r*_th_ is the ratio for setting the threshold and the standard deviation multiplied by *r*_th_ is the threshold. In this study, *r*_th_ was set to 5. Therefore, the measured data in frame *k* were excluded as outliers when the norm of the difference vector between ***z***(*k*) and ***a***(*k*), which was the estimated value of the Kalman filter, was more than five times the norm of the standard deviation vector of the tip position estimated by the Kalman filter. In this method, as the value of the right-hand side is variable, it has the advantage that an appropriate threshold can always be set according to the movement of the tip position. In principle, once an outlier is identified, if it does not return to the normal value based on Eq 22, the estimated value of the tip position ***a***(*k*) exhibits an iso-accelerated linear motion. Therefore, the filter was reset by initializing all state variables to zero when an outlier or missing value was continuously measured for more than 5 s.

### 5.2 Interpolation and smoothing method

#### 5.2.1 Interpolation process

In this study, interpolation was performed on the missing values, and the data were excluded using the aforementioned outlier removal process. Linear interpolation was used for instrument tip positions, and Slerp was used for the orientation obtained in the quaternion form.

#### 5.2.2 Smoothing process

A Savitzky–Golay filter [20] was used to calculate the tip position, smoothed velocity, acceleration, and jerk. The filter window size was set to 121 and the polynomial order was set to 3. The orientation was converted from quaternions to Z-Y-X Euler angles before the filter was applied.

#### 5.2.3 Data removal process for long-term/long-distance interpolated values

**Fig 9:**
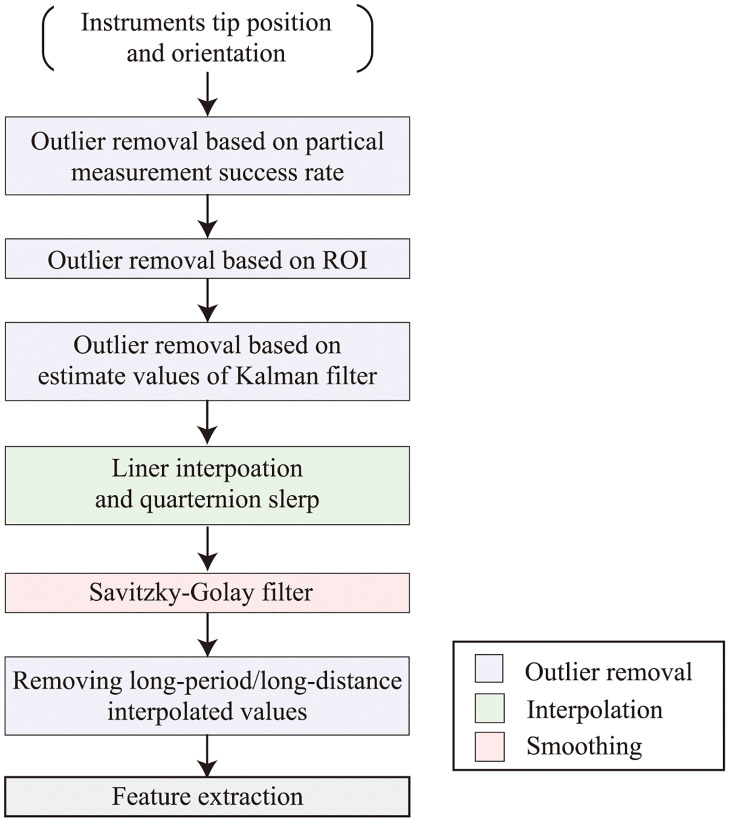
Data flow of data correction process.

**Fig 10:**
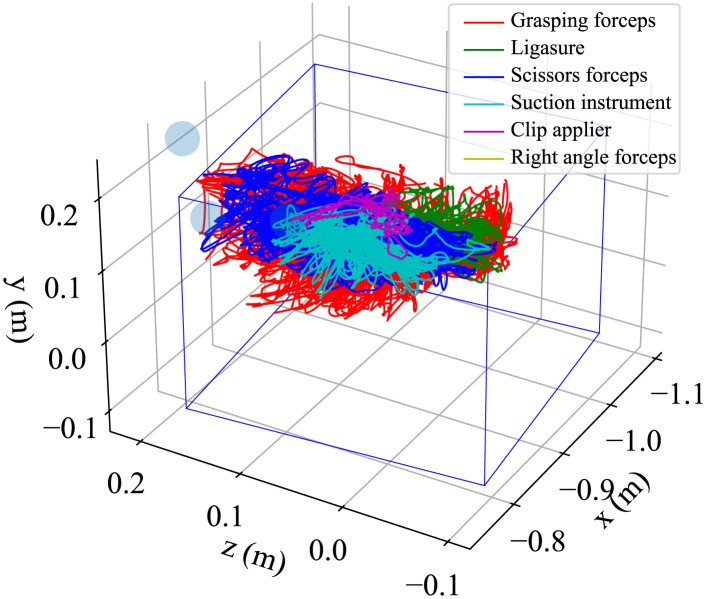
Trajectory of the tip position of all surgical instruments.

**Fig 11:**
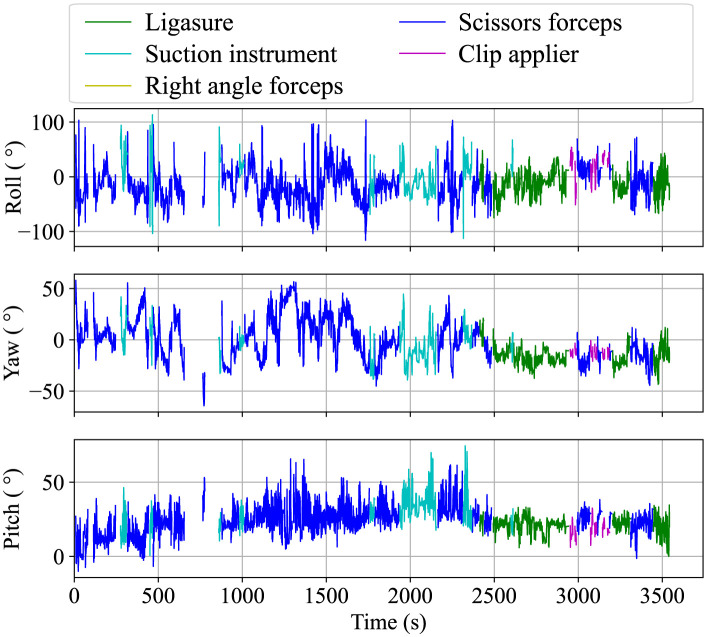
Attitude angle of the instruments handled by right hand.

Although the missing values were interpolated using the aforementioned process, the interpolated values may deviate from the actual motion of the surgical instrument, particularly when the interpolation is performed over a long period. Such data may lead to incorrect results when skill analysis is performed. Therefore, a thresholding process was used to exclude unreliable data with long interpolated values or distances. The threshold of the interpolated period was set to 5 s and that of the interpolated distance was set to 0.1 m. If the interpolated values exceeded these thresholds, they were excluded as missing values and were not used in the skill analysis. Fig 9 shows the flow of the measurement data correction process. Fig 10 shows the 3D plot of the tip positions of the surgical instruments obtained through these processes, and Fig 11 shows an example of the attitude angle of instruments handled by the surgeon’s right hand. The blue rectangular box in Fig 10 indicates the ROI and the switching of line colors in Figs 10 and 11 show the intraoperative exchange of instruments.

### 5.3 Feature extraction

The kinematic indices that reflected the surgeons’ skills in the training were calculated preliminarily from the measurement data of the 25 participants that were successfully measured. The indices were calculated for the grasping forceps, scissor forceps, and Ligasure, which were primarily used in the training task. The calculated indices were as follows:

(a) Average velocity: $\bar{v}\left(\text{cm}/\text{s}\right)$
(b) Average acceleration: $\bar{a}\left(\text{cm}/{\text{s}}^{2}\right)$
(c) Average jerk: $\bar{j}\left(\text{cm}/{\text{s}}^{3}\right)$
(d) Path length: PL (m)
(e) Depth path length: DPL (m)
(f) Average roll: ${\bar{\theta }}_{\text{roll}}$ (°)
(g) Average pitch: ${\bar{\theta }}_{\text{pitch}}$ (°)
(h) Average yaw: ${\bar{\theta }}_{\text{yaw}}$ (°)

(a)–(c) show the averages of the values calculated using the Savitzky–Golay filter. (d) shows the sum of the movement of the tip position of the instrument, (e) shows the movement along the depth direction, and (f)–(h) show the average attitude angles. These indices were calculated in the authors’ previous study for skill evaluation [15–17]. All missing frames were excluded from the calculations. Table 6 lists the results of feature extraction.

**Table 6:** Result of the feature extraction.

	Grasping forceps	Scissors forceps	Ligasure (Vessel sealing)
$\bar{v}\left(\text{cm}/\text{s}\right)$	2.02 (1.78–2.47)	2.83 (2.38–3.15)	2.89 (2.30–3.30)
$\bar{a}\left(\text{cm}/{\text{s}}^{2}\right)$	5.09 (4.48–6.46)	7.80 (6.37–8.80)	7.63 (6.22–8.93)
$\bar{j}\left(\text{cm}/{\text{s}}^{3}\right)$	34.53 (29.90–44.14)	54.01 (42.49–64.91)	52.29 (42.23–63.05)
PL (**m**)	89.17 (67.14–119.87)	62.91 (42.45–77.02)	27.98 (22.28–53.24)
DPL (**m**)	43.30 (30.61–54.74)	31.63 (22.50–42.33)	18.03 (13.78–30.34)
${\bar{\theta }}_{\text{roll}}$ (°)	1.53 (−34.08–23.86)	16.25 (−12.15–28.03)	−12.74 (−34.23–−1.18)
${\bar{\theta }}_{\text{pitch}}$ (°)	31.51 (25.10–38.27)	30.64 (22.21–39.96)	29.06 (23.26–46.54)
${\bar{\theta }}_{\text{yaw}}$ (°)	−16.76 (−28.48–−10.51)	19.84 (10.97–27.37)	21.67 (−6.37–35.49)

Median (1st quartile–3rd quartile)

## 6 Discussion

This study developed a surgical instrument motion measurement system for use in practical laparoscopic surgery training, such as cadaver surgical training. As mentioned before, the measurement system for wet-lab laparoscopic training has been developed, and a skill evaluation model has been established by calculating the kinematic indices of surgical instruments. Although the developed system can perform measurements with high accuracy, it requires a large installation space and cannot be used in practical surgical training, presenting several obstacles. Therefore, a simple motion-capture-based measurement system was developed, and the measurement accuracy was validated via a measurement experiment using animal organs and human cadavers in this study.

In the accuracy verification test described in Section 3, the measurement success rate, positional error, and attitude angle error were verified by comparing the measurement data of the proposed system (OptiTrack V120: Trio) with those of the previously developed 6-camera system (OptiTrack Prime41) in wet-lab training. As presented in Table 1, the measurement success rate of OptiTrack V120: Trio was approximately 90%, except for the right needle holder. However, the statistical analysis results show that the measurement success rate of the proposed system was significantly lower than that of the 6-camera system in all instruments. This is because of the simple configuration of OptiTrack V120: Trio, which measures the instrument with only one unit, whereas the 6-camera system performs the measurement using six cameras. The missing value of the motion data of the instruments was linearly interpolated, the median of the positional error of the task was approximately 2 mm to 4 mm, and that of the attitude angles was approximately 0.8 to 1.4°. In addition, the kinematic indices for evaluating the skills of the surgeons were calculated using the measurement data of both systems and verified by calculating Spearman’s rank correlation coefficients. As presented in Table 2, the absolute values of the correlation coefficients of all the kinematic indices exceed 0.7, and the coefficients of most of the indices exceed 0.9. This result indicates that the developed system can evaluate the skills of surgeons to the same extent as the 6-camera system. Furthermore, as presented in Table 3, regarding the operational feel of the surgical instruments, the mean evaluation values of all surgical instruments were less than 2, indicating that the surgical instruments with markers developed in this study slightly affect the operation of the instruments. These results show that although the accuracy of the developed system was slightly lower than that of the 6-camera measurement system, practical surgical measurements can be realized with the former.

In the measurement experiment in cadaver surgical training discussed in Section 4, six surgical instruments in motion were recorded for 25 cases of laparoscopic radical left/right nephrectomy. As presented in Table 4, the measurement success rate of all the surgical instruments used in the experiments was approximately 90%. Furthermore, as presented in Table 5, the mean evaluation values of the operational feel of the surgical instruments range from 1.31 to 2.03, suggesting that the instruments with markers developed in this study slightly affect the surgeries. This result suggests that adequate measurements can be performed for skill analysis during practical surgical training. As mentioned before, measurements with 6-camera systems could not be performed because of the presence of obstacles. In addition, because of the different measurement environments, the measurement data could not be compared with those acquired in the wet-lab training discussed in Section 3. Therefore, the measurement success rate of cadaver surgical training could not be evaluated in comparison with 6-camera systems. This is considered as a limitation of this study.

As mentioned earlier, because this system only measures the instrument motion from one direction, the measurement data of cadaver surgical training often include noise and outliers because of misrecognition by the MoCap system. Therefore, a data processing method for removing noise and outliers was developed. Through post-processing of the measurement data, the trajectory of the tip position (Fig 10) and attitude angle (Fig 11) were obtained. As shown in these figures, this system can identify each surgical instrument and detect intraoperative instrument changes. Therefore, the proposed system was not compared with 6-camera systems. However, these figures confirm that outliers were eliminated and reasonable measurements were performed. Table 6 presents the calculated kinematic indices of the surgical instruments for skill evaluation. The skill analysis results based on these indices will be investigated in future studies.

Other limitations of this study include a small sample size and heterogeneity, including the differing dominant hands of the subjects. As noted earlier, the measurement accuracy of the proposed system was not evaluated in cadaver surgical training because measurements using 6-camera systems could not be performed due to presence of obstacles. The effect of the low measurement success rate on skill evaluation was acceptable by the accuracy verification test in the wet-lab training. However, as presented in Table 4, approximately 10% of the data were missing in this system. This is a major challenge in using this system for other applications such as the surgical assistant system. A possible solution to this challenge is to install an inertial measurement unit (IMU) on the surgical instrument and interpolate the missing data using sensor fusion. When used alone, an IMU produces considerable errors due to the yaw angle drift. However, when employed with the MoCap system, their mutual limitations can be offset, and robust measurement can be performed. Such a measurement system is currently under development by our research group and will be reported in the future. Future studies will include skill analysis by applying other statistical analysis methods and calculating new kinematic indices of surgical instruments based on the collected data, as well as the establishment of practical surgical skill assessment methods and their application to surgical education.

## 7 Conclusion

In this study, the development and accuracy validation of a surgical instrument motion measurement system for skill evaluation during practical laparoscopic surgery training were described. The results of the accuracy verification test showed that the proposed system can measure the movement of instruments with practical accuracy and can be used for skill evaluation. By conducting measurement experiments during surgical training using cadavers, it was confirmed that the proposed system was capable of making reliable measurements with minimal impact on surgery. A limitation of this study is that the data of about 10% with long-term deficits are still not available for skill analysis. One way to address this problem is considered to compensate for missing values by sensor fusion using measurement methods other than motion capture such as IMU.

Future research involving skill analyses using the collected data is warranted. The establishment of a practical surgical skill evaluation method and its application in surgical education for the efficient acquisition of surgical skills will be considered in future studies.

## Supporting information

S1 Fig Box plots of measurement success rate in accuracy verification test. (a) Grasping forceps (b) Clip applier (c) Right angle forceps (d) Left needle holder.
(PDF)

S2 Fig Box plot of measurement success rate in cadaver surgical training. (PDF)

S1 Table Participants background in wet-lab training using porcine cadaver organ. (PDF)

S2 Table Participants background in cadaver surgical training. (PDF)
