# Supplementary figures and images for: A surgical instrument motion measurement system for skill evaluation in practical laparoscopic surgery training
Source: PLoS One. 2024 Jun 25;19(6):e0305693. doi: 10.1371/journal.pone.0305693 (PMC11198862; doi:10.1371/journal.pone.0305693)

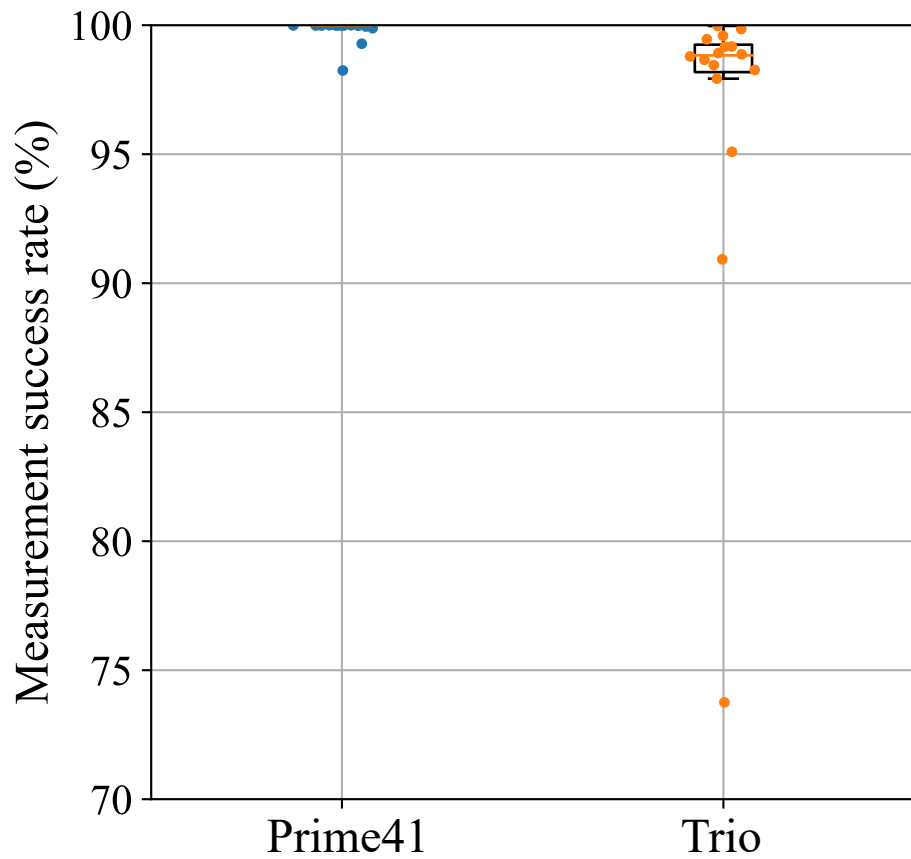

(a) Grasping forceps

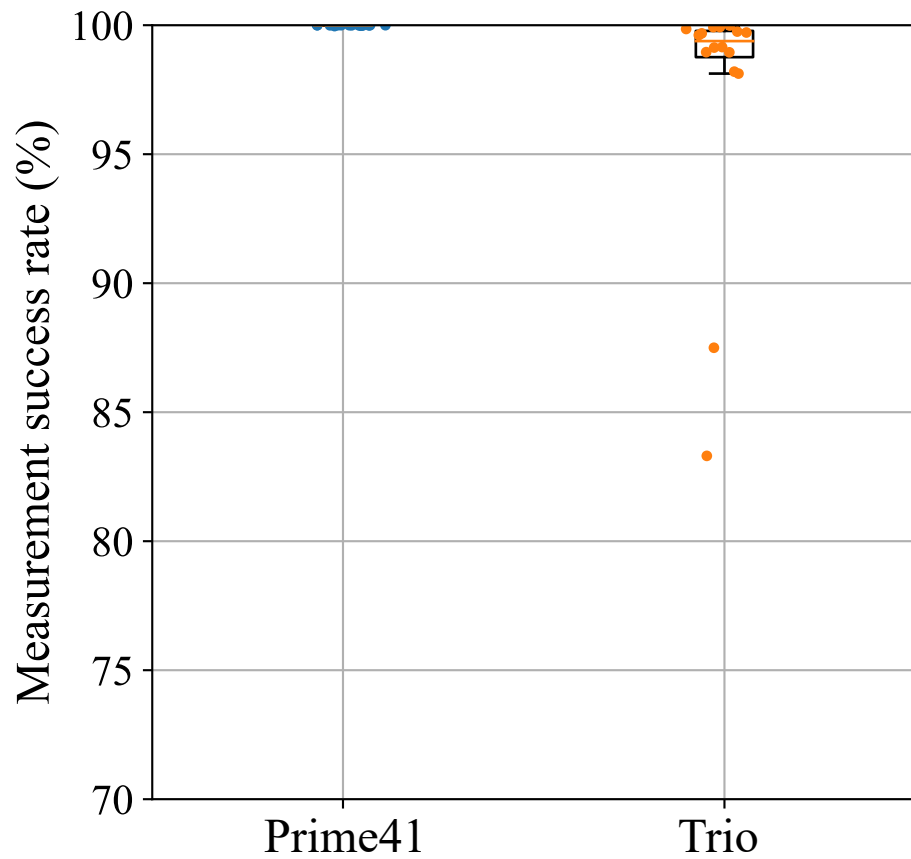

### (b) Clip applier

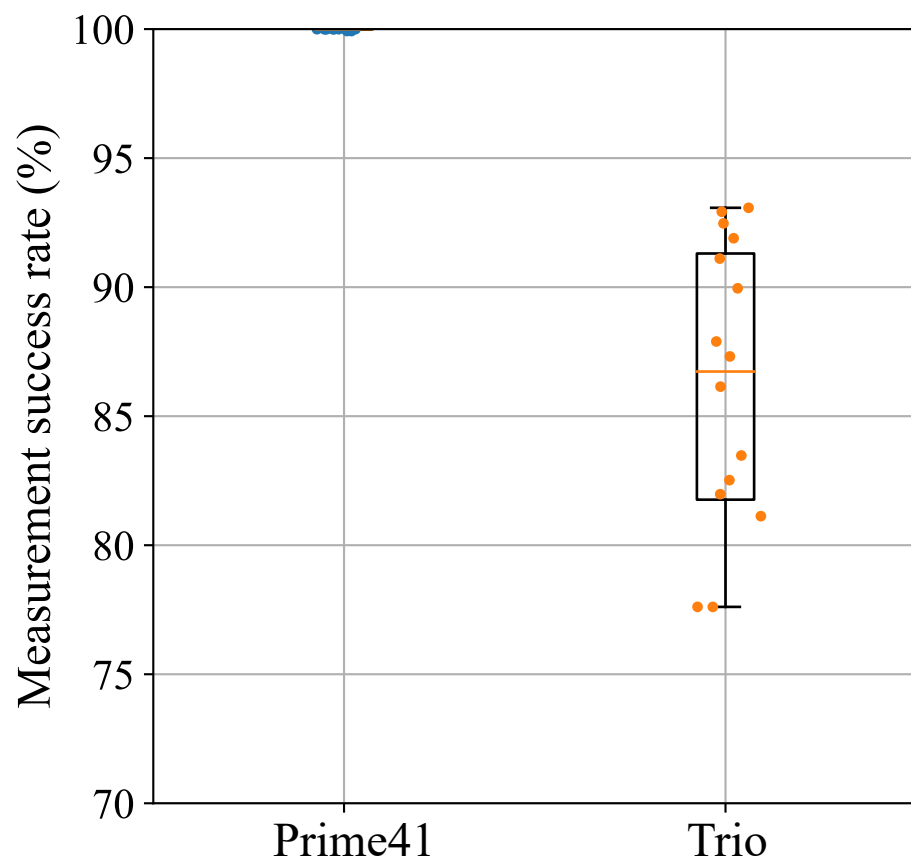

(c) Right needle holder

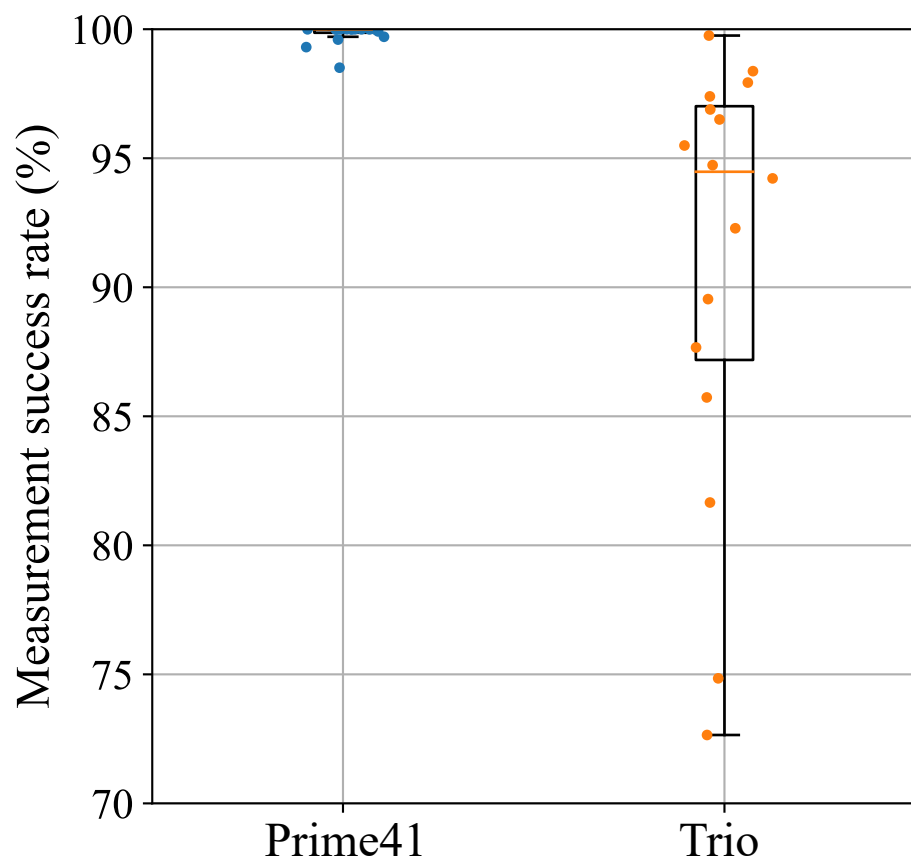

(d) Left needle holder

Supplement: S1 Fig — (a) Grasping forceps (b) Clip applier (c) Right angle forceps (d) Left needle holder. (PDF) [file pone.0305693.s001.pdf]

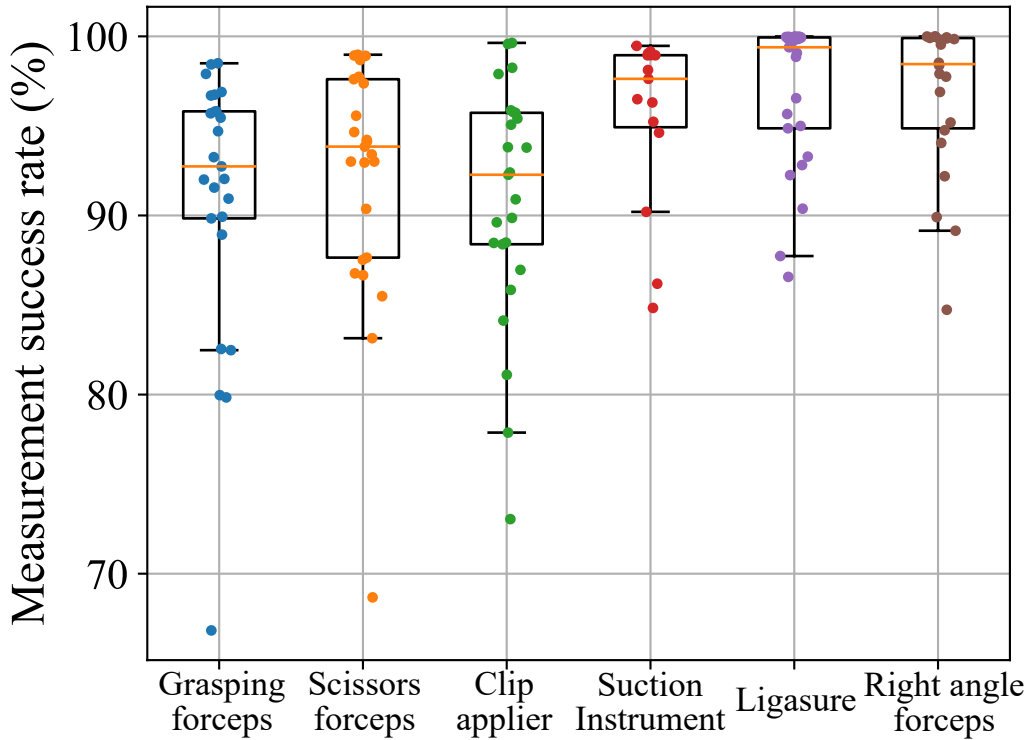

Supplement: S2 Fig — (PDF) [file pone.0305693.s002.pdf]
