# Supplementary material for: A surgical instrument motion measurement system for skill evaluation in practical laparoscopic surgery training
Source: PLoS One. 2024 Jun 25;19(6):e0305693. doi: 10.1371/journal.pone.0305693 (PMC11198862; doi:10.1371/journal.pone.0305693)
Supplement: S1 Table — (PDF) [file pone.0305693.s003.pdf]

	**n=15**
**Background**	Urologic surgeon, n=13<br>Junior resident, n=2
**Age, years**	Median 32 (range, 26-49)
**Sex**	Male/Female=13/2
**Experience of laparoscopic surgery**	0-9, n=2<br>10-49, n=5<br>50-99, n=5<br>100-499, n=2<br>≥500, n=1
**Dominant hand**	Right/left=14/1
