# Supplementary material for: A surgical instrument motion measurement system for skill evaluation in practical laparoscopic surgery training
Source: PLoS One. 2024 Jun 25;19(6):e0305693. doi: 10.1371/journal.pone.0305693 (PMC11198862; doi:10.1371/journal.pone.0305693)
Supplement: S2 Table — (PDF) [file pone.0305693.s004.pdf]

	n=31
**Background**	Urologic surgeon, n=31
**Age, years**	Median 33 (range, 27-49)
**Sex**	Male/Female=26/5
**Experience of laparoscopic surgery**	0-9, n=9<br>10-49, n=16<br>50-99, n=3<br>100-499, n=3<br>≥ 500, n=0
**Dominant hand**	Right/left=30/1
